# Editorial for Special Issue “Gangliosides: Modes of Action and Cell Fates”

**DOI:** 10.3390/ijms21186552

**Published:** 2020-09-08

**Authors:** Koichi Furukawa

**Affiliations:** Department of Life and Biomedical Sciences, Chubu University College of Life and Health Sciences, Matsumoto 1200, Kasugai, Aichi 487-8501, Japan; koichi@med.nagoya-u.ac.jp

**Keywords:** gangliosides, synthesis, degradation, neurodegeneration, aging, glycosyltransferase, glycosphingolipids

## Abstract

Gangliosides have been considered to play essential roles in the regulation of nervous systems. Novel findings about their functions based on the unique genetic and biochemical approaches have been recently accumulated, and representative results were collected here. In particular, new developments of analytical methods, regulatory mechanisms for ganglioside synthesis and degradation, and novel aspects of their functions in nervous systems and various other organs were introduced in this Special Issue, promoting further fundamental investigation and applied research.

Since Thudichum reported the glycolipids in 1884, information about them have been gradually expanded. In particular, the discovery of a representative accumulative disease of GSLs in lysosomes based on the deficiency of their degradation enzymes, Tay-Sach’s disease, by Klenk has made us recognize the presence and significance of glycosphingolipids in our bodies [1]. However, major issues which researchers face have been pathological effects of accumulated GSLs in CNS and also in all over bodies [2,3]. On the other hand, the characterization of chemical structures of newly-defined GSLs and the biological meaning of individual species of GSLs have been investigated by Yamakawa and Nagai [4], Wiegandt [2], Tettamanti [5], Hakomori [6], and many other researchers. 

Gangliosides, sialic acid-containing glycosphingolipids, are ubiquitously expressed mainly on the cell membrane in vertebrates. The structure of carbohydrate moieties varies depending on the situations under which tissues and cells exist. In particular, the polymorphic alteration of gangliosides along with the development and differentiation of nervous tissues has suggested their roles in the organogenesis of nervous systems [7,8]. In turn, some gangliosides have been considered to be cancer-associated antigens, and have been used as tumor markers and targets for antibody therapy [6,9,10,11].

Since cDNAs for ganglioside synthases were isolated [12], the genetic modification of ganglioside expression in cultured cells and experimental animals has enabled us to substantially elucidate the roles of gangliosides in our bodies. Based on the following understandings, i.e., (i) Gangliosides play a pivotal role in the maintenance of the integrity of nervous systems, and the repair of damaged nerve tissues; (ii) Gangliosides are important for the architecture and function of membrane microdomains; and (iii) Gangliosides regulate cell signals at lipid rafts based on the interaction with ganglioside-recognizing molecules, leading to the decision of cell fates, the Special Issue called for original researches and reviews and perspectives that address the progress and current knowledge in the research on gangliosides. Consequently, a number of manuscripts have been submitted, and 13 papers (seven original papers and six review articles) of a high quality and with a strong impact have been published as summarized below.

Our goal is to clarify the puzzle of polymorphic structures of gangliosides, and to reveal the mechanisms for molecular interactions in which gangliosides are involved, and finally demonstrate the consequences of those actions in vitro and in vivo. Although a number of ganglioside species have been defined to date, new structures and novel functions of them have been searched. Cavdarli et al. elucidated the expression of 9-*O*-acetyl forms of GD2 in various cancer cells, and found crucial roles of them particularly in cancer cells [13]. Although 9-*O*-acetyl forms of gangliosides have been reported from a long time ago, actual properties of them have not been well understood. Cavdarli’s paper would give us an insight. In order to understand the molecular functions of gangliosides, precise structure analysis is essential. Miyazaki et al. developed a unique method to distinguish fine differences in sialylation linkages that are frequently critical in the recognition of sialylated carbohydrates with ligand molecules [14]. They showed a nice example of its application for the evaluation of residual iPS cells in human chondrocytes. Asano’s group developed a unique technique to label Gb3, Gb4 and globo-H as well as many gangliosides using fluorescence dye [15]. These compounds are very useful to analyze the behaviors of GSLs on the membrane and/or inside of cells, and also to observe the interaction of GSLs with other membrane molecules, lectins, and the cell itself. Mojumdar et al. developed a new system for the analysis of self-assembly in the ganglioside-phospholipid system [16]. This system is very useful to analyze actual behaviors of GSLs present with many phospholipids on the cell membrane. All four of these articles should contribute to the fundamental approaches for the investigation of “Modes of Action of Gangliosides”.

For the basis of the synthesis and degradation of gangliosides, three intriguing papers were published, i.e., ganglioside synthesis by plasma membrane-associated sialyltransferase [17], dietary control of ganglioside expression in mammalian tissues [18], and secondary gangliosides and lipid accumulation in lysosomal disease [3]. Although the majority of glycosyltransferases are considered to localize in Golgi, Vilcaes et al. showed that a sialyltransferase exists in the plasma membrane of macrophages. Actual roles of the membrane-associated sialyltransferase may open a new concept of sialyltransferases, particularly in inflammation [17]. If ganglioside compositions expressed on cells and tissues can be controlled by foods, novel approaches may become possible for application in health care and ganglioside-related diseases [18]. Breiden’s group has long studied various gangliosidosis. Interestingly, in addition to the accumulation of direct substrates of the degradation enzymes, secondary gangliosides such as GM2 and other lipids have been known to accumulate [3] in many lysosomal storage diseases, leading to neurodegeneration [3].

As a consequence of ganglioside action, novel findings in various events of biological and pathological processes were observed, and six interesting papers were published. First of all, gangliosides in spermatozoa, oocytes, and preimplantation embryos were reported [19], showing functional roles of gangliosides in the reproductive system based on effects of exogenous gangliosides. Then, novel molecular mechanisms of gangliosides in the nervous system were reviewed based on the findings from genetically engineered animals and cells [20]. Among gangliosides, GM1 has been considered to be a key factor in maintaining the mammalian neuronal functions [21]. The roles of GM1 have been most rigorously analyzed due to its unique functions in neurogenesis and neurodegeneration, and Chiricozzi et al. proposed its application for neuroprotection. Unique neuroprotective activity of Neurotropin due to crosstalk of neurotrophic and innate immune receptors was also reported by Fukuda et al. [22]. Gangliosides in vascular diseases have also shown an interesting theme [23], and their roles in endothelial cells, vascular-associated cells, and inflammatory cells for atherosclerosis and aging were reported. Finally, little is known about roles of GD3 synthase and its products in bone metabolism. Yo et al. clearly demonstrated implication of gangliosides in bone resorption using GD3 synthase-KO mice [24] as a novel function. 

All 12 articles have brought a new wind in the field of ganglioside research based on unique approaches on the individual aspects, and should promote further challenges especially of young researchers. However, there may be a lot of issues to be solved to understand the enigma of GSLs. For example, we do not know much about regulatory systems for ganglioside synthesis and degradation, and those for the complex formation of gangliosides and other membrane molecules on a molecular basis [25]. Future-enriched information on these points would be the basis for the actual application of ganglioside functions.
